# Supplementary material for: Clinical Considerations for Patients Experiencing Acute Kidney Injury Following Percutaneous Nephrolithotomy
Source: Biomedicines. 2023 Jun 14;11(6):1712. doi: 10.3390/biomedicines11061712 (PMC10296554; doi:10.3390/biomedicines11061712)
Supplement: Supplementary file 1 [file biomedicines-11-01712-s001.zip › biomedicines-2421865-supplementary.pdf]

Supplementary Materials

Table S1. Detailed patient characteristics by AKI occurrence and trajectories.

			AKI			Rapidly Reversed AKI			P-AKI	
	No AKI	AKI	AKI Stage 1	AKI Stage 2+	Rapid Reversed AKI	Mild Rapid Reversed AKI	Severe Rapid Reversed AKI	P-AKI	Mild P-AKI	Severe P-AKI
Number of patients, n	254	93	79	18	71	66	5	27	15	13
Number of encounters, n	313	104	85	19	75	70	5	29	15	14
Demographic information										
Age, years, mean (SD)	53 (15)	58 (16)[a]	58 (17)[b]	55 (14)	59 (16)[d]	58 (16)[e]	64 (7)	54 (16)	57 (17)	51 (15)
Sex, n (%)										
Female, n (%)	153 (49)	51 (49)	45 (53)	6 (32)	36 (48)	35 (50)	1 (20)	15 (52)	10 (67)	5 (36)
Body Mass Index, median (IQR)	29 (25, 33)	29 (24, 35)	29 (24, 35)	29 (25, 40)	28 (24, 34)	28 (24, 34)	28 (28, 29)	33 (27, 40)	33 (27, 39)	31 (25, 41)
Race, n (%)										
African American	26 (8)	14 (13)	12 (14)	2 (11)	10 (13)	9 (13)	1 (20)	4 (14)	3 (20)	1 (7)
Other	53 (17)	10 (10)	8 (9)	2 (11)	8 (11)	7 (10)	1 (20)	2 (7)	1 (7)	1 (7)
White	234 (75)	80 (77)	65 (76)	15 (79)	57 (76)	54 (77)	3 (60)	23 (79)	11 (73)	12 (86)
Ethnicity, n (%)										
Hispanic	19 (6)	6 (6)	5 (6)	1 (5)	5 (7)	5 (7)	0 (0)	1 (3)	0 (0)	1 (7)
Marital Status, n (%)										
Married	152 (49)	50 (48)	40 (47)	10 (53)	39 (52)	35 (50)	4 (80)	11 (38)	5 (33)	6 (43)
Single	122 (39)	37 (36)	29 (34)	8 (42)	22 (29)	21 (30)	1 (20)	15 (52)	8 (53)	7 (50)
Divorced	34 (11)	17 (16)	16 (19)	1 (5)	14 (19)	14 (20)	0 (0)	3 (10)	2 (13)	1 (7)
Native Language Spoken, n (%)										
Non-English, n (%)	10 (3)	4 (4)	3 (4)	1 (5)	3 (4)	3 (4)	0 (0)	1 (3)	0 (0)	1 (7)
Insurance paying the bills, n (%)										
Medicare	109 (35)	60 (58)[a]	49 (58)[b]	11 (58)	44 (59)[d]	42 (60)[e]	2 (40)	16 (55)[g]	7 (47)	9 (64)
Private	121 (39)	26 (25)[a]	19 (22)[b]	7 (37)	19 (25)	16 (23)	3 (60)	7 (24)	3 (20)	4 (29)
Medicaid	53 (17)	12 (12)	12 (14)	0 (0)	9 (12)	9 (13)	0 (0)	3 (10)	3 (20)	0 (0)
Uninsured	30 (10)	6 (6)	5 (6)	1 (5)	3 (4)	3 (4)	0 (0)	3 (10)	2 (13)	1 (7)
Smoking Status, n (%)										
Never	165 (53)	49 (47)	37 (44)	12 (63)	35 (47)	31 (44)	4 (80)	14 (48)	6 (40)	8 (57)
Former	76 (24)	37 (36)	31 (36) [b]	6 (32)	26 (35)	25 (36)	1 (20)	11 (38)	6 (40)	5 (36)
Current	65 (21)	18 (17)	17 (20)	1 (5)	14 (19)	14 (20)	0 (0)	4 (14)	3 (20)	1 (7)
Missing	7 (2)	0 (0)	0 (0)	0 (0)	0 (0)	0 (0)	0 (0)	0 (0)	0 (0)	0 (0)
Distance of residence to hospital, miles, mean (SD)	45 (49)	43 (32)	26 (19)	31 (21)	42 (30)	25 (18)	36 (17)	46 (36)	29 (22)	29 (22)
Rural at patient residential area, n (%)	106 (34)	27 (26)	19 (22)	8 (42)	19 (25)	17 (24)	2 (40)	8 (28)	2 (13)	6 (43)
Total population at patient residential area, mean (SD)	17922 (11558)	18802 (11119)	18867 (11205)	18510 (10725)	18084 (10535)	18054 (10560)	18500 (10158)	20659 (12313)	22661 (13173)	18514 (10920)

Median total income at patient residential area, USD, mean (SD)	41644 (11189)	40136 (10050)	39755 (9836)	41840 (10792)	39634 (9769)	39348 (9676)	43642 (10182)	41432 (10632)	41652 (10341)	41197 (10930)
Prevalence of residents living below poverty in patient residential area, %, mean (SD)	20.37 (8.51)	21.00 (9.18)	21.56 (9.57)	18.49 (6.60)	21.41 (9.68)	21.81 (9.82)	15.76 (4.31)	19.95 (7.65)	20.39 (8.20)	19.47 (6.99)
Prevalence of African American residents living below poverty at patient residential area, %	0.18 (0.17)	0.19 (0.18)	0.20 (0.19)	0.16 (0.16)	0.19 (0.18)	0.20 (0.19)	0.15 (0.11)	0.18 (0.19)	0.20 (0.20)	0.16 (0.17)
Prevalence of Hispanic residents living below poverty at patient residential area, %	0.08 (0.08)	0.09 (0.06)	0.09 (0.07)	0.08 (0.04)	0.09 (0.06)	0.09 (0.06)	0.11 (0.05)	0.09 (0.06)	0.09 (0.07)	0.08 (0.03)
Time from Admission to Surgery, days, median (IQR)	0 (0, 0)	0 (0, 2)	0 (0, 2)	0 (0, 2)[c]	0 (0, 2)	0 (0, 2)	0 (0, 0)	0 (0, 2)	0 (0, 2)	0 (0, 6)
Emergency admission	23 (7)	10 (10)	7 (8)	3 (16)	4 (5)	4 (6)	0 (0)	6 (21)	3 (20)	3 (21)
The admission happened at night	121 (39)	40 (38)	33 (39)	7 (37)	25 (33)	24 (34)	1 (20)	15 (52)	9 (60)	6 (43)
Weekend admission	12 (4)	4 (4)	4 (5)	0 (0)	4 (5)	4 (6)	0 (0)	0 (0)	0 (0)	0 (0)
Transferred from another hospital	7 (2)	1 (1)	1 (1)	0 (0)	1 (1)	1 (1)	0 (0)	0 (0)	0 (0)	0 (0)
Reference estimated glomerular filtration rate, median (IQR)	84.93 (67.81, 103.32)	85.70 (44.08, 103.43)	80.06 (44.08, 102.97)	92.88 (56.79, 103.23)	79.72 (41.19, 102.69)	79.89 (42.23, 104.60)	36.03 (29.57, 62.51)	92.60 (61.64, 103.23)	88.73 (58.42, 97.23)	93.58 (67.81, 103.82)
CPT Code										
50080 - PCNL ≤ 2cm, n (%)	23 (7)	10 (10)	8 (9)	2 (11)	9 (12)	8 (11)	1 (20)	1 (3)	0 (0)	1 (7)
SOFA score, median (IQR)	0.00 (0.00, 0.00)	0.00 (0.00, 5.00)[a]	0.00 (0.00, 5.00)[b]	0.00 (0.00, 2.00)	0.00 (0.00, 4.00)	0.00 (0.00, 4.00)	0.00 (0.00, 0.00)	0.00 (0.00, 8.00)[g]	0.00 (0.00, 7.50)	0.00 (0.00, 7.50)
ASA score, median (IQR)	3.00 (2.00, 3.00)	3.00 (3.00, 3.00)[a]	3.00 (3.00, 3.00)	3.00 (3.00, 3.25)	3.00 (3.00, 3.00)	3.00 (3.00, 3.00)	NA	3.00 (3.00, 3.50)	3.00 (2.50, 3.50)	3.00 (3.00, 3.25)

[a]: Bonferroni adjusted p-value ≤ 0.05 for AKI vs No AKI comparison.
[b]: Bonferroni adjusted p-value ≤ 0.05 for AKI Stage 1 vs No AKI comparison.
[c]: Bonferroni adjusted p-value ≤ 0.05 for AKI Stage 2+ vs No AKI comparison.
[d]: Bonferroni adjusted p-values ≤ 0.05 for Rapidly Reversed AKI vs No AKI comparison.
[e]: Bonferroni adjusted p-values ≤ 0.05 for Mild Rapidly Reversed AKI vs No AKI comparison.
[f]: Bonferroni adjusted p-values ≤ 0.05 for Severe Rapidly Reversed AKI vs No AKI comparison.
[g]: Bonferroni adjusted p-values ≤ 0.05 for P-AKI vs No AKI comparison.
[h]: Bonferroni adjusted p-values ≤ 0.05 Mild P-AKI vs No AKI comparison.
[i]: Bonferroni adjusted p-values ≤ 0.05 for Severe P-AKI vs No AKI comparison.

Abbreviations: AKI, Acute kidney injury; ASA, American Society of Anesthesiology; CPT, Current Procedural Terminology; IQR, Interquartile range; P-AKI, Persistent acute kidney injury; PCNL, Percutaneous Nephrolithotomy; SD, standard deviation; SOFA, Sequential Organ Failure Assessment.

Table S2. Medical comorbidities with regard to AKI.

			AKI			Rapidly Reversed AKI			P-AKI	
	**No AKI**	**AKI**	**AKI Stage 1**	**AKI Stage 2+**	**Rapid Reversed AKI**	**Mild Rapid Reversed AKI**	**Severe Rapid Reversed AKI**	**P-AKI**	**Mild P-AKI**	**Severe P-AKI**
Charlson comorbidity index, median (IQR)	1 (0, 3)	2 (0, 5)[a]	2 (0, 4)[b]	3 (1, 6)[c]	2 (0, 5)[d]	2 (0, 5)[e]	4 (3, 6)	3 (0, 5)[g]	2 (0, 3)	3 (0, 5)
Alcohol or drug abuse	18 (6)	8 (8)	4 (5)	4 (21)	3 (4)	3 (4)	0 (0)	5 (17)	1 (7)	4 (29)[i]
Myocardial Infarction	14 (4)	5 (5)	3 (4)	2 (11)	4 (5)	3 (4)	1 (20)	1 (3)	0 (0)	1 (7)
Congestive Heart Failure	26 (8)	12 (12)	9 (11)	3 (16)	8 (11)	7 (10)	1 (20)	4 (14)	2 (13)	2 (14)
Peripheral Vascular Disease	25 (8)	19 (18)[a]	15 (18)[b]	4 (21)	13 (17)	13 (19)	0 (0)	6 (21)	2 (13)	4 (29)
Cerebrovascular Disease	19 (6)	5 (5)	3 (4)	2 (11)	3 (4)	3 (4)	0 (0)	2 (7)	0 (0)	2 (14)
Chronic Pulmonary Disease	68 (22)	32 (31)	23 (27)	9 (47)	22 (29)	18 (26)	4 (80)	10 (34)	5 (33)	5 (36)
Cancer	43 (14)	23 (22)	18 (21)	5 (26)	17 (23)	15 (21)	2 (40)	6 (21)	3 (20)	3 (21)
Metastatic Carcinoma	11 (4)	9 (9)	8 (9)	1 (5)	8 (11)	7 (10)	1 (20)	1 (3)	1 (7)	0 (0)
Liver Disease	34 (11)	11 (11)	8 (9)	3 (16)	7 (9)	7 (10)	0 (0)	4 (14)	1 (7)	3 (21)
Diabetes	63 (20)	29 (28)	26 (31)	3 (16)	21 (28)	20 (29)	1 (20)	8 (28)	6 (40)	2 (14)
Hypertension	33 (11)	19 (18)[a]	17 (20)	2 (11)	15 (20)	14 (20)	1 (20)	4 (14)	3 (20)	1 (7)
Obesity	74 (24)	33 (32)	25 (29)	8 (42)	19 (25)	18 (26)	1 (20)	14 (48)[g]	7 (47)	7 (50)
Fluid and electrolyte disorders	40 (13)	36 (35)[a]	28 (33)[b]	8 (42)[c]	24 (32)[d]	23 (33)[e]	1 (20)	12 (41)[g]	5 (33)	7 (50)[i]
Valvular Disease	15 (5)	15 (14)	9 (11)	6 (32)[c]	7 (9)	7 (10)	0 (0)	8 (28)[g]	2 (13)	6 (43)[i]
Coagulopathy	12 (4)	6 (6)	3 (4)	3 (16)	4 (5)	3 (4)	1 (20)	2 (7)	0 (0)	2 (14)
Weight Loss	19 (6)	16 (15)[a]	14 (16)[b]	2 (11)	12 (16)[d]	12 (17)	0 (0)	4 (14)	2 (13)	2 (14)
Depression	32 (10)	13 (12)	12 (14)	1 (5)	10 (13)	10 (14)	0 (0)	3 (10)	2 (13)	1 (7)
Chronic anemia	14 (4)	13 (12)[a]	11 (13)[b]	2 (11)	8 (11)	8 (11)	0 (0)	5 (17)[g]	3 (20)	2 (14)
Chronic Kidney Disease	76 (24)	46 (44)[a]	38 (45)[b]	8 (42)	34 (45)[d]	31 (44)[e]	3 (60)	12 (41)	7 (47)	5 (36)

[a]: Bonferroni adjusted p-value ≤ 0.05 for AKI vs No AKI comparison.
[b]: Bonferroni adjusted p-value ≤ 0.05 for AKI Stage 1 vs No AKI comparison.
[c]: Bonferroni adjusted p-value ≤ 0.05 for AKI Stage 2+ vs No AKI comparison.
[d]: Bonferroni adjusted p-values ≤ 0.05 for Rapidly Reversed AKI vs No AKI comparison.
[e]: Bonferroni adjusted p-values ≤ 0.05 for Mild Rapidly Reversed AKI vs No AKI comparison.
[f]: Bonferroni adjusted p-values ≤ 0.05 for Severe Rapidly Reversed AKI vs No AKI comparison.
[g]: Bonferroni adjusted p-values ≤ 0.05 for P-AKI vs No AKI comparison.
[h]: Bonferroni adjusted p-values ≤ 0.05 Mild P-AKI vs No AKI comparison.
[i]: Bonferroni adjusted p-values ≤ 0.05 for Severe P-AKI vs No AKI comparison.
Abbreviations: AKI, Acute kidney injury; P-AKI, Persistent acute kidney injury.

Table S3. Preoperative medication exposures within 30 days of surgery with regard to AKI.

			AKI			Rapidly Reversed AKI			P-AKI	
	**No AKI**	**AKI**	**AKI Stage 1**	**AKI Stage 2+**	**Rapid Reversed AKI**	**Mild Rapid Reversed AKI**	**Severe Rapid Reversed AKI**	**P-AKI**	**Mild P-AKI**	**Severe P-AKI**
Aminoglycosides, n (%)	109 (35)	35 (34)	30 (35)	5 (26)	28 (37)	26 (37)	2 (40)	7 (24)	4 (27)	3 (21)
Analgesics, n (%)	204 (65)	67 (64)	55 (65)	12 (63)	49 (65)	46 (66)	3 (60)	18 (62)	9 (60)	9 (64)
Angiotensins, n (%)	15 (5)	6 (6)	6 (7)	0 (0)	5 (7)	5 (7)	0 (0)	1 (3)	1 (7)	0 (0)
Antifungals, n (%)	42 (13)	24 (23)[a]	20 (24)	4 (21)	17 (23)	17 (24)	0 (0)	7 (24)	3 (20)	4 (29)
Beta Blockers, n (%)	57 (18)	25 (24)	23 (27)	2 (11)	20 (27)	20 (29)	0 (0)	5 (17)	3 (20)	2 (14)
Beta Lactams, n (%)	13 (4)	4 (4)	4 (5)	0 (0)	3 (4)	3 (4)	0 (0)	1 (3)	1 (7)	0 (0)
Cephalosporins, n (%)	88 (28)	34 (33)	27 (32)	7 (37)	21 (28)	20 (29)	1 (20)	13 (45)	7 (47)	6 (43)
Corticosteroids, n (%)	40 (13)	27 (26)[a]	22 (26)[b]	5 (26)	20 (27)[d]	20 (29)[e]	0 (0)	7 (24)	2 (13)	5 (36)
Diuretics, n (%)	43 (14)	14 (13)	10 (12)	4 (21)	9 (12)	9 (13)	0 (0)	5 (17)	1 (7)	4 (29)
Fluoroquinoles, n (%)	13 (4)	4 (4)	4 (5)	0 (0)	3 (4)	3 (4)	0 (0)	1 (3)	1 (7)	0 (0)
Heparins, n (%)	112 (36)	46 (44)	38 (45)	8 (42)	33 (44)	31 (44)	2 (40)	13 (45)	7 (47)	6 (43)
Nonsteroidal anti-inflammatory drugs, n (%)	59 (19)	15 (14)	12 (14)	3 (16)	10 (13)	10 (14)	0 (0)	5 (17)	2 (13)	3 (21)
Salicylates, n (%)	35 (11)	9 (9)	7 (8)	2 (11)	7 (9)	5 (7)	2 (40)	2 (7)	2 (13)	0 (0)
Statins, n (%)	13 (4)	4 (4)	4 (5)	0 (0)	3 (4)	3 (4)	0 (0)	1 (3)	1 (7)	0 (0)
Penicillins, n (%)	127 (41)	38 (37)	34 (40)	4 (21)	30 (40)	27 (39)	3 (60)	8 (28)	7 (47)	1 (7)
Vasopressors, n (%)	74 (24)	20 (19)	16 (19)	4 (21)	15 (20)	14 (20)	1 (20)	5 (17)	2 (13)	3 (21)

[a]: Bonferroni adjusted p-value ≤ 0.05 for AKI vs No AKI comparison.
[b]: Bonferroni adjusted p-value ≤ 0.05 for AKI Stage 1 vs No AKI comparison.
[c]: Bonferroni adjusted p-value ≤ 0.05 for AKI Stage 2+ vs No AKI comparison.
[d]: Bonferroni adjusted p-values ≤ 0.05 for Rapidly Reversed AKI vs No AKI comparison.
[e]: Bonferroni adjusted p-values ≤ 0.05 for Mild Rapidly Reversed AKI vs No AKI comparison.
[f]: Bonferroni adjusted p-values ≤ 0.05 for Severe Rapidly Reversed AKI vs No AKI comparison.
[g]: Bonferroni adjusted p-values ≤ 0.05 for P-AKI vs No AKI comparison.
[h]: Bonferroni adjusted p-values ≤ 0.05 Mild P-AKI vs No AKI comparison.
[i]: Bonferroni adjusted p-values ≤ 0.05 for Severe P-AKI vs No AKI comparison.

Abbreviations: AKI, Acute kidney injury; P-AKI, Persistent acute kidney injury.

Table S4. Univariate odds ratios tables for AKI stages and trajectories.

	No AKI vs AKI		Stage 1 AKI vs Stage 2+ AKI		Rapidly Reversed AKI vs P-AKI	
	**OR (95% CI)**	**p-value**	**OR (95% CI)**	**p-value**	**OR (95% CI)**	**p-value**
Sex (Reference: Female)						
Male	0.99 (0.64, 1.55)	>0.9	2.44 (0.88, 7.50)	0.10	0.86 (0.36, 2.04)	0.7
Race (Reference: White)						
African-American	1.57 (0.77, 3.12)	0.2	0.72 (0.11, 3.03)	0.7	0.99 (0.25, 3.30)	>0.9
Other	0.55 (0.25, 1.09)	0.11	1.08 (0.15, 4.89)	>0.9	0.62 (0.09, 2.71)	0.6
Ethnicity (Reference: Non-Hispanic)						
Hispanic	0.95 (0.34, 2.31)	>0.9	1.08 (0.15, 4.89)	>0.9	0.50 (0.03, 3.29)	0.5
Payer (Reference: Medicaid)						
Medicare	2.43 (1.24, 5.09)	0.013	NA	NA	1.09 (0.28, 5.37)	>0.9
Private	0.95 (0.45, 2.08)	0.9	NA	NA	1.11 (0.24, 6.05)	>0.9
Uninsured	0.88 (0.28, 2.52)	0.8	NA	NA	3.00 (0.38, 26.5)	0.3
Marital Status (Reference: Single)						
Divorced	1.65 (0.82, 3.26)	0.2	0.23 (0.01, 1.40)	0.20	0.31 (0.06, 1.17)	0.11
Married	1.08 (0.67, 1.77)	0.7	0.91 (0.32, 2.64)	0.9	0.41 (0.16, 1.05)	0.065
Admission emergency (Reference: Non-emergency)						
Emergency	1.34 (0.59, 2.85)	0.5	2.09 (0.42, 8.44)	0.3	4.63 (1.22, 19.5)	0.026
Smoking status (Reference: Never)						
Current	0.93 (0.50, 1.70)	0.8	0.18 (0.01, 1.04)	0.11	0.71 (0.18, 2.41)	0.6
Former	1.64 (0.99, 2.72)	0.056	0.60 (0.19, 1.73)	0.4	1.06 (0.41, 2.70)	>0.9
Rural (Reference: Urban)	0.68 (0.41, 1.11)	0.13	2.53 (0.87, 7.18)	0.082	1.12 (0.41, 2.89)	0.8
CPT 50081 (Reference: 50080)	0.75 (0.35, 1.69)	0.5	0.88 (0.20, 6.20)	0.9	3.82 (0.67, 72.1)	0.2
Alcohol or drug abuse (Reference: No)	1.37 (0.55, 3.14)	0.5	5.40 (1.16, 25.2)	0.027	5.00 (1.14, 25.9)	0.036
Myocardial Infarction (Reference: No)	1.08 (0.34, 2.90)	0.9	3.22 (0.40, 20.9)	0.2	0.63 (0.03, 4.52)	0.7
Congestive Heart Failure (Reference: No)	1.44 (0.68, 2.91)	0.3	3.22 (0.40, 20.9)	0.5	1.34 (0.33, 4.66)	0.7
Peripheral Vascular Disease (Reference: No)	2.58 (1.34, 4.89)	0.004	1.24 (0.32, 4.02)	0.7	1.24 (0.40, 3.56)	0.7
Cerebrovascular Disease (Reference: No)	0.78 (0.25, 2.00)	0.6	3.22 (0.40, 20.9)	0.2	1.78 (0.22, 11.3)	0.5
Chronic Pulmonary Disease (Reference: No)	1.60 (0.97, 2.62)	0.063	2.43 (0.86, 6.79)	0.088	1.27 (0.50, 3.13)	0.6
Cancer (Reference: No)	1.78 (1.00, 3.11)	0.044	1.33 (0.39, 4.01)	0.6	0.89 (0.29, 2.45)	0.8
Metastatic Carcinoma (Reference: No)	2.60 (1.02, 6.47)	0.040	1.33 (0.39, 4.01)	0.6	0.30 (0.02, 1.74)	0.3
Liver Disease (Reference: No)	0.97 (0.45, 1.94)	>0.9	1.80 (0.37, 7.04)	0.4	1.55 (0.38, 5.61)	0.5
Diabetes (Reference: No)	1.53 (0.91, 2.54)	0.10	0.43 (0.09, 1.42)	0.2	0.98 (0.36, 2.50	>0.9
Hypertension (Reference: No)	1.90 (1.01, 3.48)	0.041	0.47 (0.07, 1.86)	0.3	0.64 (0.17, 1.97)	0.5
Obesity (Reference: No)	1.50 (0.91, 2.44)	0.10	0.47 (0.07, 1.86)	0.3	2.75 (1.12, 6.81)	0.027

Fluid and electrolyte disorders (Reference: No)	3.61 (2.14, 6.11)	<0.001	1.48 (0.52, 4.08)	0.4	1.50 (0.61, 3.63)	0.4
Valvular Disease (Reference: No)	3.35 (1.57, 7.17)	0.002	3.90 (1.14, 12.8)	0.025	1.31 (0.18, 7.15)	0.8
Coagulopathy (Reference: No)	1.54 (0.52, 4.07)	0.4	5.12 (0.88, 30.0)	0.058	0.84 (0.22, 2.68)	0.8
Weight Loss (Reference: No)	2.81 (1.37, 5.70)	0.004	0.60 (0.09, 2.41)	0.5	0.75 (0.16, 2.69)	0.7
Depression (Reference: No))	1.25 (0.61, 2.44)	0.5	0.34 (0.02, 1.89)	0.3	1.74 (0.49, 5.76)	0.4
Chronic anemia (Reference: No)	3.05 (1.37, 6.76)	0.006	0.79 (0.12, 3.31)	0.8	2.14 (0.90, 5.18)	0.087
Chronic Kidney Disease (Reference: No)	2.47(1.55, 3.94)	<0.001	0.90 (0.32, 2.45)	0.8	0.85 (0.35, 2.02)	0.7
Night admission (Reference: No)	0.99 (0.63, 1.56	>0.9	0.79 (0.12, 3.31)	0.9	2.14 (0.90, 5.18)	0.087
Aminoglycosides (Reference: No)	0.99 (0.61, 1.59)	>0.9	0.79 (0.12, 3.31)	0.3	0.47 (0.17, 1.19)	0.13
Analgesics (Reference: No)	1.06 (0.65, 1.74)	0.8	0.29 (0.04, 1.11)	0.6	0.67 (0.27, 1.69)	0.4
Angiotensins (Reference: No)	1.26 (0.44, 3.19)	0.6	NA	NA	0.46 (0.02, 3.01)	0.5
Antifungals (Reference: No)	2.02 (1.14, 3.54)	0.014	0.29 (0.04, 1.11)	0.7	0.97 (0.34, 2.61)	>0.9
Beta Blockers (Reference: No)	1.48 (0.86, 2.52)	0.2	0.29 (0.04, 1.11)	0.11	0.51 (0.16, 1.44)	0.2
Beta Lactams (Reference: No)	0.95 (0.26, 2.77)	>0.9	NA	NA	0.79 (0.04, 6.44)	0.8
Cephalosporins (Reference: No)	1.30 (0.80, 2.11)	0.3	1.12 (0.38, 3.13)	0.8	1.86 (0.76, 4.56)	0.2
Corticosteroids (Reference: No)	2.51 (1.43, 4.36)	0.001	0.93 (0.27, 2.75)	0.9	0.78 (0.27, 2.05)	0.6
Diuretics (Reference: No)	0.98 (0.50, 1.83)	>0.9	2.00 (0.50, 6.91)	0.3	1.53 (0.43, 4.90)	0.5
Fluoroquinoles (Reference: No)	0.95 (0.26, 2.77)	>0.9	NA	NA	0.79 (0.04, 6.44)	0.8
Heparins (Reference: No)	1.52 (0.96, 2.40)	0.076	0.78 (0.28, 2.15)	0.6	0.89 (0.37, 2.12)	0.8
Nonsteroidal anti-inflammatory drugs (Reference: No)	0.75 (0.39, 1.36)	0.4	1.05 (0.22, 3.78)	>0.9	1.23 (0.35, 3.85)	0.7
Salicylates (Reference: No)	0.78 (0.34, 1.61)	0.5	1.05 (0.22, 3.78)	0.8	0.66 (0.09, 2.93)	0.6
Statins (Reference: No)	0.95 (0.26, 2.77)	>0.9	NA	NA	0.79 (0.04, 6.44)	0.8
Penicillins (Reference: No)	0.88 (0.55, 1.40)	0.6	0.35 0.09, 1.07	0.086	0.50 (0.18, 1.24)	0.14
Vasopressors (Reference: No)	0.80 (0.45, 1.37)	0.4	1.05 (0.22, 3.78)	>0.9	0.75 (0.22, 2.19)	0.6

Abbreviations: AKI, Acute kidney injury; CI, Confidence Interval; P-AKI, Persistent acute kidney injury.

Table S5. Univariate analysis for hospital length of stay.

	Estimate (95% CI)	Standard Error	p-value
Sex (Reference: Female)			
Male	-0.61 (-1.59, 0.36)	0.5	0.22
Age	0.01 (-0.03, 0.04)	0.02	0.72
BMI	0.03 (-0.03, 0.08)	0.03	0.31
Charlson Comorbidity Index	0.35 (0.16, 0.53)	0.09	0
Race (Reference: White)			
African-American	0.17 (-1.49, 1.83)	0.85	0.84
Other	-1.79 (-3.15, -0.42)	0.7	0.01
Payer (Reference: Medicaid)			
Medicare	0.57 (-0.85, 1.99)	0.72	0.43
Private	-1.88 (-3.33, -0.43)	0.74	0.01
Uninsured	-1.41 (-3.44, 0.61)	1.03	0.17
Ethnicity (Reference: Non-Hispanic)			
Hispanic	-0.07 (-2.12, 1.98)	1.05	0.95
Marital Status (Reference: Single)			
Divorced	1.27 (-0.31, 2.85)	0.81	0.11
Married	-1.33 (-2.37, -0.29)	0.53	0.01
Missing	-1.68 (-6.14, 2.78)	2.27	0.46
Admission emergency (Reference: Non-emergency)			
Emergency	6.57 (4.88, 8.27)	0.86	0
Smoking Status (Reference: Never)			
Current	0.03 (-1.26, 1.31)	0.66	0.97
Former	1.15 (-0.01, 2.3)	0.59	0.05
Missing	0.18 (-3.64, 3.99)	1.95	0.93
Alcohol or drug abuse (Reference: No)	4.56 (2.59, 6.53)	1	0
Congestive Heart Failure (Reference: No)	0.09 (-2.25, 2.43)	1.19	0.94
Cerebrovascular Disease (Reference: No)	1.72 (0.03, 3.41)	0.86	0.05
Cancer (Reference: No)	2.96 (1.4, 4.53)	0.8	0
Liver Disease (Reference: No)	2.19 (0.1, 4.27)	1.06	0.04
Hypertension (Reference: No)	1.33 (0.2, 2.46)	0.58	0.02
Fluid and electrolyte disorders (Reference: No)	-1.25 (-2.58, 0.08)	0.68	0.07
Coagulopathy (Reference: No)	-1.45 (-3.73, 0.83)	1.16	0.21
Depression (Reference: No))	1.2 (-0.37, 2.76)	0.8	0.13
Chronic Kidney Disease (Reference: No)	0.93 (-0.24, 2.1)	0.6	0.12
Myocardial Infarction (Reference: No)	1.07 (-0.4, 2.54)	0.75	0.16
Peripheral Vascular Disease (Reference: No)	1.95 (0.85, 3.05)	0.56	0
Chronic Pulmonary Disease (Reference: No)	4.69 (3.51, 5.87)	0.6	0
Metastatic Carcinoma (Reference: No)	6.81 (5.05, 8.58)	0.9	0
Diabetes (Reference: No)	3.11 (0.73, 5.49)	1.21	0.01
Obesity (Reference: No)	2.84 (1.1, 4.57)	0.89	0
Valvular Disease (Reference: No)	2.21 (0.65, 3.76)	0.79	0.01
Weight Loss (Reference: No)	5.2 (3.28, 7.12)	0.98	0
Chronic anemia (Reference: No)	1.31 (0.25, 2.38)	0.54	0.02
AKI			
AKI vs No AKI	4.83 (3.8, 5.86)	0.52	0

Worst AKI Stage			
AKI Stage 1 vs No AKI	3.5 (2.5, 4.5)	0.51	0
AKI Stage 2 vs No AKI	6.66 (4.43, 8.88)	1.14	0
AKI Stage 3 vs No AKI	22.31 (18.63, 25.99)	1.88	0
CPT (Reference: 50080)			
50081	0.35 (0.16, 0.53)	0.09	0

Abbreviations: AKI, Acute kidney injury; CI, Confidence Interval; P-AKI, Persistent acute kidney injury.

TableS6. Odds ratios for the adjusted logistic regression model for AKI.

	**Odds Ratio (95% CI)**	**p-value**
Age	1.02 (1.00, 1.04)	0.014
Charlson Comorbidity Index	1.08 (0.98, 1.20)	0.13
Chronic Kidney Disease		
No	—	
Yes	1.75 (0.96, 3.18)	0.066
Race		
White	—	
White vs African-American	1.34 (0.59, 2.93)	0.5
White vs Other	0.38 (0.15, 0.85)	00.028
Corticosteroid		
No	—	
Yes	3.19 (1.72, 5.94)	<0.001

Abbreviations: CI, Confidence Interval.

Table S7. Outcomes of hospital length of stay regression model.

	**Beta**	**p-value**
Age	-0.02 (-0.05, 0.01)	0.3
Sex		
Female	-	
Male	-0.65 (-1.6, 0.27)	0.2
Race		
White	-	
African-American	-0.63 (-2.2, 0.94)	0.4
Other	-1.2 (-2.4, 0.13)	0.08
Body mass index	0.01 (-0.04, 0.06)	0.8
Charlson Comorbidity Index	0.24 (0.03, 0.45)	0.023
Chronic Kidney Disease		
No	-	
Yes	-0.26 (1.5, 0.94)	0.7
AKI		
No	-	
Yes	4.9 (3.8, 5.9)	<0.001

Abbreviations: AKI, Acute kidney injury.

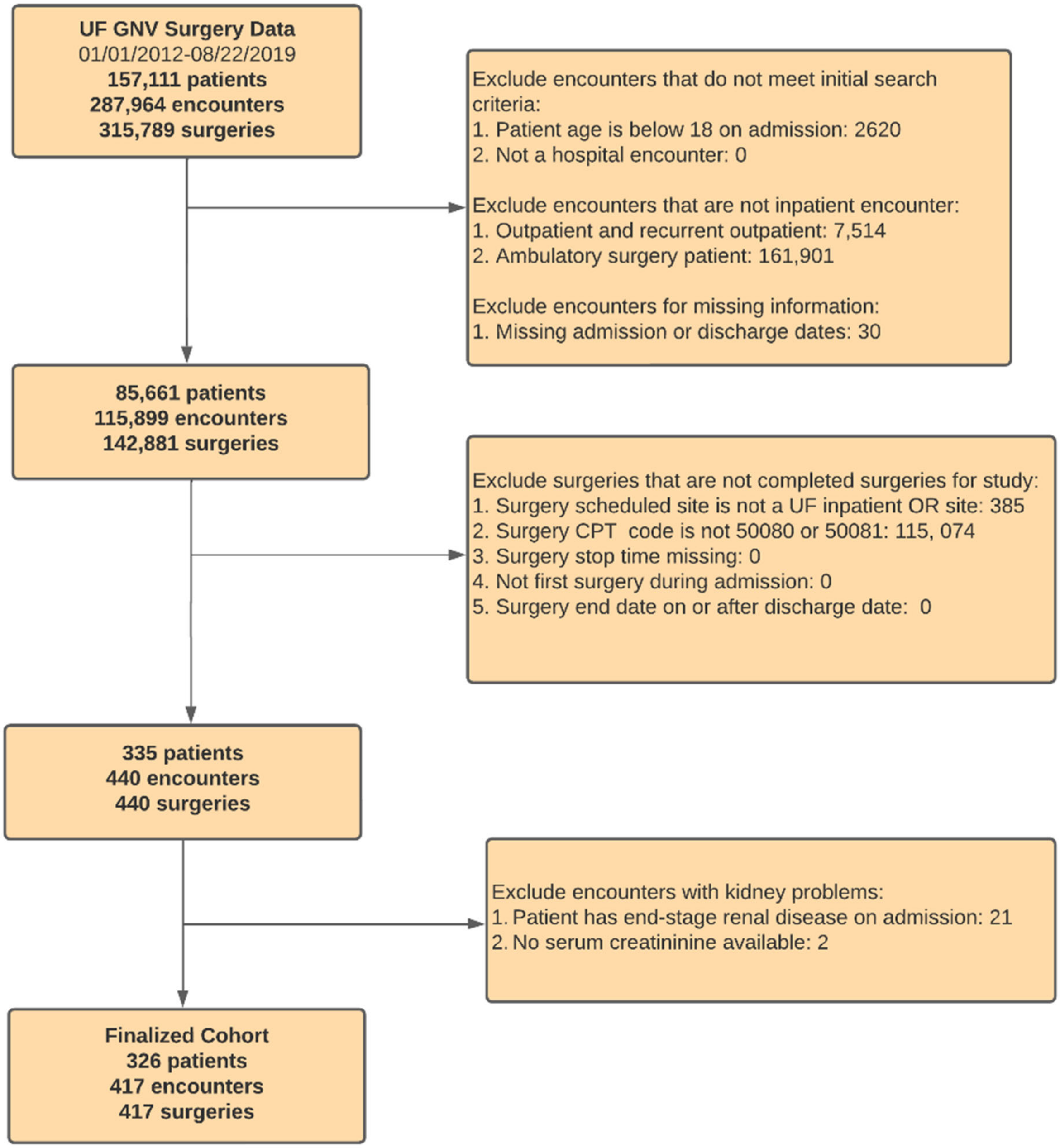

Figure S1. Cohort derivation.
